# Carbon Footprint of Plastic Bags and Polystyrene Dishes vs. Starch-Based Biodegradable Packaging in Amazonian Settlements

**DOI:** 10.3390/polym17243242

**Published:** 2025-12-05

**Authors:** Johanna Garavito, Néstor C. Posada, Clara P. Peña-Venegas, Diego A. Castellanos

**Affiliations:** 1 Food Packaging and Shelf Life Laboratory, Instituto de Ciencia y Tecnología de Alimentos, Universidad Nacional de Colombia, Carrera 30 Número 45-03, Bogotá 111321, Colombia; ngaravitoj@unal.edu.co (J.G.); nposada@unal.edu.co (N.C.P.); 2 Instituto Amazónico de Investigaciones Científicas–SINCHI, Avenida Vásquez Cobo Calle 15/16, Leticia 910001, Colombia; cpena@sinchi.org.co

**Keywords:** single-use packaging, sustainability, biodegradability, carbon emission, plastic waste

## Abstract

C footprint is a feature used to search the integral life cycle of a product to predict its environmental impact. The packaging industry is changing rapidly to the production of biodegradable products to mitigate the negative environmental consequences of the use of single-use packages. It is thought that biodegradable packages should be more sustainable than traditional plastics due to the sources of the raw materials used to produce them, but this is not always true and depends on the issues considered, the methodology, and the scale analyzed. Limited research includes case studies from developing countries where waste management is less efficient and where the environmental impacts of single-use packaging can be more significant. This paper evaluates the C footprint of bags and dishes made from traditional or local biodegradable sources in an Amazonian settlement of Colombia, such as thermoplastic cassava starch and powdered plantain leaves, to evaluate the impact of locally made biodegradable packaging vs. imported petrochemical ones. Results show that using local raw materials and in situ production reduces the C footprint of biodegradable packages, considering that the energy source for production and transport are important contributors to the C footprint beyond the raw materials used, with ratios that can be between 0.1 and 7 times more kg CO_2_ eq generated per functional unit.

## 1. Introduction

Since the beginning of plastic production, around 8300 million tons of plastic have been produced [1]. From them, 40% of plastic is used for the packaging industry, where most of it is produced from new raw materials; only around 7% of plastic is recycled. As the food industry continues using plastic as the primary raw material for all types of food packaging, plastic continues to increase the total global footprint daily.

The global plastic footprint includes macroplastics produced by mismanaged waste disposal processes and microplastics generated from the first one, but these cannot be removed easily from where they are present. The plastic footprint includes around 4.5% of global greenhouse gas (GHG) emissions [2], plastic incineration, and plastic leakage that remains in landfills and natural environments. The discovery that microplastics can be bio-accumulated, causing alteration in chromosomes related to infertility, obesity, and cancer, and that could also affect other living organisms [3,4], is another of the biggest concerns today to replace plastics. Some preliminary initiatives include policies for restricting and banning the use of traditional plastics and searching for more sustainable alternatives, including the production of bio-packaging alternatives and products into a circular economy model.

Several biodegradable polymers are used in this field, most of them derived from natural resources and capable of decomposing under controlled conditions. For example, polylactic acid (PLA) is a biodegradable polyester produced by fermenting plant sugars (such as corn starch) [5]. PLA is compostable under controlled industrial conditions where temperature and humidity favor its complete conversion to water and CO_2_. Another example is polyhydroxyalkanoates (PHAs), polymers produced by microorganisms from biomass; these materials are fully biodegradable even in natural environments (soil and aquatic environments) [6]. Starch-based plastics are also used; for example, thermoplastic starch derived from corn or cassava, which, when combined with appropriate plasticizers and additives, generates renewable, non-toxic, and compostable biopolymers [7]. Furthermore, in the case of disposable tableware, lignocellulosic materials such as molded sugarcane bagasse pulp have been developed, whose fibers allow for the manufacture of biodegradable and compostable plates. These bagasse plates, after use, can disintegrate in a matter of weeks under composting conditions, leaving no toxic residues or microplastics [8,9].

Single-use plastics such as plastic bags, meal boxes, and cups comprise approximately half of the annual plastic production, which was close to 360–400 million tons for 2020 [10,11]. In response to this, single-use biodegradable packaging has been promoted as one feasible alternative to cope with plastic contamination. Replacing fossil fuels as the main raw material for single-packaging production with biomass might reduce emissions and achieve an absolute reduction from the current level of plastics contamination [12]. However, calculations from China and the USA indicated that biodegradable packaging could not always reduce the carbon footprint when compared with that produced from fossil fuel sources [13]. In most cases, it must be with the issue that organic matter produces a complete decomposition, producing at the end between 10 and 90% more CO_2_, which contributes to GHG emissions; plastics can be produced with less amount of energy and might require less carbon consumption for their transportation to be lighter than other biodegradable packaging.

Although several studies have inventoried and quantified different sources of plastic leakage either nationally [14,15], internationally [16,17], or from specific riverine environments [18], the values obtained depend on the methodology, model, and data used. For example, plastic leakage at the global level has been estimated between 8 and 12.2 Mt [19]. However, those global estimations include many assumptions to obtain their estimations that could not reflect reality. According to Boucher et al. [19], these methodologies lack data on plastic waste management at the country level, pathway models accounting for both microplastic and macroplastic leakage, fate models about how they are transferred to the environment, and impact assessment to account for the negative effects of plastics on human health and ecosystems. Fair comparisons must include fossil-based vs. bio-based and non-compostable vs. compostable at the life cycle assessment (LCA) of each product [20]. But the main limitation of this kind of study is the need for data to calculate the life cycle assessment of materials [21]. It is supposed that bio-based materials can perform better than fossil-based ones when footprints are compared, but it will depend on the way they are evaluated [22], making it difficult to assess their real impact. Thus, specific rather than global comparisons could help to provide more realistic information about product footprints.

The most used footprint methodologies proposed to quantify plastic footprint include the following: (1) inventories of plastics leaked into the environment; (2) the impact on human health; and (3) the valuation of the cost of externalities from plastic leaking. Those methodologies could be adjusted to compare fossil-based and biodegradable plastics, providing an opportunity to compare the footprints of the two types of materials. The scale of leakage of plastics and their C footprint depends on the type of package and the geographical context in which the life cycle of the package occurs [17]. Until today, there is limited information about the carbon footprint estimation of single-use packages in developing countries, where there are more limitations in good waste disposal and recycling, and where the environmental implications could significantly affect naturally well-preserved ecosystems. Although net GHG emissions from plastics could be less than those from biomass, few studies include the evaluation of the carbon source. It is not the same C from an almost permanent carbon storage as fossil fuels as from biomass, which is a C already in the biosphere, with a transitory carbon storage as biomass, and with zero net increase in biospheric carbon stocks. Additionally, plastics are not easily degradable and, therefore, are more stable. Bio-based products have a lower feedstock carbon emission burden than fossil-based products. The reduction in plastic permanence in the environment is difficult, and that is why it is associated with an increased risk to human and environmental health.

This paper aims to provide a local study of the environmental impact of two biodegradable packages produced locally based in cassava starch and plantain leaves vs. petrochemical plastic packages commercialized locally, estimating the carbon footprint and the residues generated in the end of life in each case, which could provide a reliable comparison of those two types of packages based on primary information obtained in the study area.

## 2. Materials and Methods

### 2.1. Study Site

**Figure 1:**
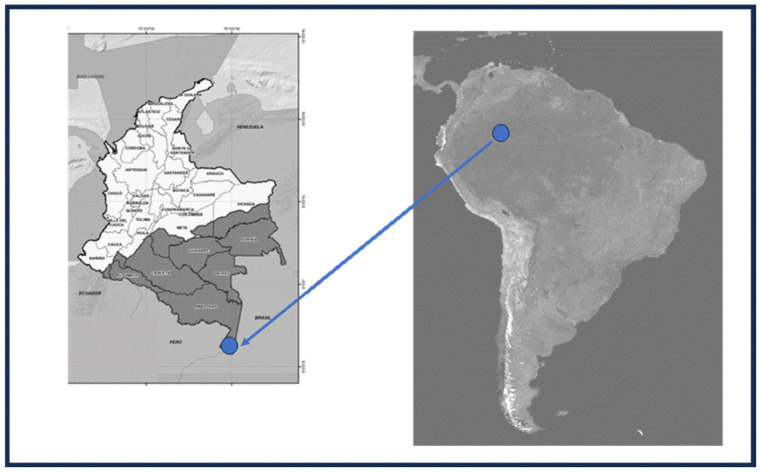
Map of the study site.

The estimations performed in the study about C footprint and plastic waste degradation were obtained for the Amazon region and specifically from two municipalities of the Southern Colombian Amazon: Leticia and Puerto Nariño, which are located on the northern side of the Amazon River (Figure 1).

### 2.2. Functional Units Evaluated

A functional unit (FU) is defined as the quantified performance of a product system used as a reference in an LCA [23]. Two types of plastic packaging were evaluated: (1) petrochemical-based packaging consisting of 1 kg low-density polyethylene (LDPE) bags, and 20 × 25 cm wide polyethylene terephthalate (PET) plates with a capacity of 12 ounces (0.34 kg); (2) biodegradable bags with a 1 kg capacity and biodegradable plates measuring 10 × 7 cm developed by the Amazonian Institute for Scientific Research (SINCHI) on the frame of the project BIOEMPAQUES BPIN No. 20180001000062 “Desarrollo de bioempaques a partir de recursos amazónicos renovables Amazonas” [Biopacking developing from natural renewal resources from the Amazon], and produced locally in Puerto Nariño, Department of Amazonas, Colombia. Regarding biodegradable packaging in (2), both the bags and the dishes were made with a base mixture of thermoplastic cassava starch (TPS-cassava) and powdered plantain leaves, in addition to additives such as glycerol and beeswax [24]. For the bags, the starch content was 71%, and the plantain leaves content was 1% (both *w*/*w*), while for the plates, it was 32% and 13%, respectively. In addition, the bags used also contained 2% (*w*/*w*) low-density polyethene (LDPE) in their composition [24]. Estimates were based on 1000 units per package as the functional unit (FU) for this study.

### 2.3. Footprint Estimation Approach

Based on the LCA of the two bags and the two dishes, four different steps were studied to calculate, in each one, the quantity of C involved: (1) the raw materials used; (2) the product production process; (3) the transport required to access the market (or user); and (4) the waste produced.

(1) Raw Materials Enclosed: Energy for their obtention, carbon content in the final material of each product. Cassava and plantain leaves are produced in small agricultural plots without the use of machinery or external inputs as fertilizers or insecticides. No irrigation is needed as the region has a tropical rainforest, characterized by high humidity. Due to this low input production, the average of cassava production was estimated at 3 kg/plant, according to local field estimations performed by the authors.
(2) Product Production Process Encloses: Energy and source of energy to process raw materials and manufacture the final product. Cassava starch is produced by artisanal techniques by local Indigenous people by hand or using a low 5HP gasoline motor for grating. Plantain leaves do not have any previous treatment. Dried plantain leaves are collected directly from the agricultural plots and brought to the local pilot factory.
(3) Transportation encloses emissions, which are calculated using the average distance traveled from product manufacturing facilities to downtown, where the products are sought from small stores or direct users (in terms of the cheapest transport available to reach that place).
(4) The waste produced was estimated based on the average recycling of plastic in the region. Calculations consider that only 4% of plastic is recycled, according to the regional plan for the integrated management of solid residues–PGIRS 2017 [25]. Calculations will consider the carbon remaining and the GHG emissions based on the degradability of each product (from degradability assays carried out by the Amazonian Institute for Scientific Research—SINCHI, in situ), and the recalcitrant carbon remaining in the environment and the cost that might be required for their final decomposition into non-toxic final compounds. Additionally, calculations of the consumption of the containers will be estimated, based on the direct interviews with bag distributors, triangulating their answers with interviews with commercial stores about the consumption of this particular bag per month.

### 2.4. Carbon Footprint Assessment for Raw Material Composition

The material’s carbon footprint was quantified by calculating the product of each constituent’s mass fraction and its corresponding CO_2_ emission factor, expressed in kg CO_2_-eq/kg. The emission factors were sourced from the existing literature and the CarbonCloud^®^ database [26], a comprehensive carbon data platform. The carbon footprint was then determined by summing these individual contributions, providing an indicative estimate of environmental impact based on its raw material composition. It is important to note that these values are reference estimates and may vary depending on specific life cycle stages and processing methods employed during raw material extraction and processing.

Based on the theoretical CO_2_-eq per raw-material formulation for composite TPS-cassava bags and dishes, it was possible to calculate the kg of CO_2_ emitted per functional unit (FU), considering an estimated weight per bag of 0.005 kg and per dish of 0.208 kg, respectively:

(1) $$FU\;bags=\left(0.005\;\frac{kg}{bag}\times \;1000\;bags\right)=5\;kg$$

(2) $$FU\;dishes=\left(0.208\;\frac{kg}{Dishes}\times 1000\;dishes\right)=208\;kg$$

The weight per unit used (0.005 kg per bag and 0.208 kg per dish) are higher than those of conventional plastics because the mixture of TPS, powered plantain fiber and glycerol required more raw material in order to obtain a material of appropriate mechanical strength under the local processing conditions evaluated: sheet extrusion instead of blown-film extrusion for bags, and manual or semi-manual compressión-molding instead of injection or thermoforming for dishes. Therefore, the FU was defined as kilograms of bags and kilograms of dishes to ensure consistent environmental comparison.

### 2.5. Energy Consumption and Carbon Footprint Assessment in Composite TPS-Cassava Bag Production

Energy consumption for each stage of the composite TPS-cassava bag and dishes production process was quantified based on equipment specifications and operational requirements. The total hourly energy usage was determined by aggregating the individual energy demands of all equipment involved. The carbon footprint of the production process was then calculated by combining the total energy consumption with emission factors specific to various energy sources. These emission factors, expressed in kg CO_2_-eq per kWh, were obtained from the established literature [27]. For each energy source, the total carbon emissions per hour were derived by multiplying its emission factor by the total energy consumed.

The emissions derived from energy consumption per functional unit (FU) were calculated by considering a pilot production line of 20 kg/h.

(3) $$Rate\;of\;bag\;production=\frac{20\frac{kg}{h}}{\frac{0.005kg}{bag}}=4000\frac{bags}{h}$$

$$If\;1FU=1000\;bags,\;\;\;\;rate\;of\;FU\;production=4\frac{FU}{h}$$

(4) $$Rate\;of\;dish\;production=\frac{20\frac{kg}{h}}{\frac{0.208kg}{dish}}=96.16\frac{dishes}{h}$$

$$If\;1FU=1000\;dishes,\;\;\;\;rate\;of\;FU\;production=0.096\frac{FU}{h}$$

### 2.6. Carbon Footprint Analysis of Transportation Processes

The carbon footprint of transportation for composite TPS-cassava bags and dishes production was assessed by comparing two scenarios: (i) transporting raw materials to the site of production, and (ii) transporting finished plastic bags from Bogotá. The analysis considered two transport legs: air freight (1095 km from Bogotá to Leticia) and river transport via gasoline-powered boats (80 km from Leticia to Puerto Nariño). In the local production scenario, only non-locally available raw materials, accounting for 28% of the total product mass, were transported. In contrast, in the plastic bag import scenario, the entire 10-ton batch was moved along the same route. Carbon emissions were estimated using fuel-specific emission factors, expressed in kg CO_2_ per ton-km, sourced from databases [28]. The emissions for each transport leg were computed using Equation (1).

(5) $$\begin{array}{cc} \mathrm{Carbon}\mathrm{Emissions}\; & \left(\mathrm{kg}{\mathrm{CO}}_{2}\right)\\ & =\mathrm{Distance}\;(\mathrm{km})\times \mathrm{Transported}\mathrm{Mass}\;(\mathrm{tons})\\ & \times \mathrm{Emission}\mathrm{Factor}\;(\mathrm{kg}{\mathrm{CO}}_{2}/\mathrm{ton}-\mathrm{km}) \end{array}$$

Emissions were summed across transport modes to obtain the total footprint for each scenario. For the calculation of the corresponding emissions per functional unit (FU), we assume that the transported raw material (RM) will produce 10 tons of biodegradable bags, as local inputs at the production site complement it. The process and equations used to calculate the functional CO_2_ emissions per unit (FU) of bags and dishes are described below:

10 ton=2000 FU ofbagsor48.08FUofdishes

(6) $$\frac{{kg\;of\;CO}_{2}}{FU}=\frac{Emissions\;Raw\;Materials\;transported}{Number\;of\;FU}$$

In the particular case of this biopackage production, there are no local extra transportation inputs, as Puerto Nariño is a small village without motor vehicles, where all transport is made on foot.

### 2.7. Estimation of the Carbon Footprint of Final Life of Each Product

#### 2.7.1. Biodegradability Test of Composite TPS-Cassava Bags and Dishes at Laboratory Conditions

A controlled composting test was conducted at 58 °C to evaluate the biodegradation behavior of composite TPS-cassava bags and dishes based on the ASTM D5338-15(2021) “Standard Test Method for Determining Aerobic Biodegradation of Plastic Materials Under Controlled Composting Conditions, Incorporating Thermophilic Temperatures” [29]. Square 1 × 1 cm samples were carefully cut and placed in airtight composting chambers containing a pre-conditioned humus layer moistened with distilled water to maintain optimal microbial activity. The humus composition was characterized by 13.1% organic carbon, 61.7% ash content, a pH of 6.6, and a density of 0.926 g/mL, ensuring a standardized environment for biodegradation assessment. The samples were incubated at 58 °C and 70% RH, and degradation was evaluated by monitoring mass loss and CO_2_ evolution, both key indicators of microbial activity and polymer breakdown. The cumulative CO_2_ released over time was quantified to determine the extent of biodegradation, while the percentage of CO_2_ evolved relative to the theoretical maximum provided insights into the mineralization efficiency of the cut bags and dishes. Biodegradability was expressed as the fraction of carbon from the composite TPS-cassava packaging pieces converted into CO_2_, enabling a direct assessment of their decomposition under composting conditions.

Additionally, laboratory values were compared with in situ field assays of the bags and dishes’ degradability to establish if there were statistical differences between the two, which might indicate unrealistic assumptions of degradability used for this exercise.

#### 2.7.2. Accumulation of Non-Biodegradable Residues

To evaluate the release of non-biodegradable residues, a comparative analysis was conducted between composite TPS-cassava bags and conventional plastic bags. The annual release of non-biodegradable residues was calculated using Equation (7) [30,31].

(7) $$R_{non-biodegradable}=C_{bag}\times W_{bag}\times \%non-biodegradable\times \left(1-DegradationRate\right)$$

where

*R*_non-biodegradable_: Non-biodegradable residues released annually (kg).
*C*_bag_: Annual consumption of bags (units).
W_bag_: Weight of each bag (kg).
% Non-Biodegradable: Proportion of non-biodegradable components in the material.
Degradation Rate: Annual degradation rate of non-biodegradable content (%).

As mentioned above, the composite TPS-cassava bag formulation evaluated for this study contains 2% LDPE, a non-biodegradable petroleum-based polymer, while conventional plastic bags consist entirely of non-biodegradable petrochemical polymers. The degradation rates were assumed to be 0.5% per year for conventional LDPE [32].

Finally, it is necessary to mention that the previous analysis does not consider the specific impact that the generation of microplastics could have on the environment in the analysis of the materials degradation, since a methodology for this purpose was not established in the study.

## 3. Results

### 3.1. Carbon Footprint Estimation of Each Package

The total carbon footprint from raw materials was determined solely based on their contributions to the factors of emission. A carbon footprint assessment of composite TPS-cassava biodegradable bags and dishes provides an indicative estimate of the potential emissions associated with their raw material composition (Table 1). Cassava starch is the main biodegradable material used to produce the biodegradable packages produced in Puerto Nariño. The cassava starch used to produce those packages is grown, harvested, and obtained by artisanal techniques in local Indigenous communities on agricultural subsistence plots. Then, cassava cultivation is free of external inputs such as synthetic fertilizers that could increase the C footprint of the materials [33]. Then, only C, as the main component of starch molecules, was counted. Glycerol is the second largest carbon contributor on the evaluated biodegradable formulas, with 31.3% in bags and 23.2% in dishes, with a higher carbon footprint due to its industrial production, which often involves chemical synthesis or bio-based processes such as those obtained by fermentation [34].

Carbon footprint estimations for plastic bags and dishes based on the most common materials used are shown in Table 2.

The manufacturing process for composite TPS-cassava materials requires electrical energy for every kilogram of material produced; the energy consumption data for starch-derived packages is summarized in Table 3.

Carbon emissions per hour for composite TPS-cassava bags and dishes production are summarized in Table 4.

Estimates on carbon footprint related to transportation were calculated according to the type of transport required for each bag or dish, in terms of the mobility of local materials to reach the place for their production, and the transport required to reach the point of commercialization and the final customer. Later ones are summarized in Table 5.

### 3.2. Estimation of the Carbon Footprint of End of Life of Each Product

As previously mentioned for the bags and dishes in this case study, the base components were cassava TPS, ground plantain leaves, and glycerol. The cassava starch was incorporated as the main polymer matrix, glycerol (approx. 30% *w*/*w*) was incorporated as a plasticizer [41], and the plantain fibers were added as a lignocellulosic reinforcement to increase the mechanical strength of the TPS [42,43]. Additionally, other components were used as additives to improve the functional performance of these packages under the climatic conditions in which they are used. In particular, beeswax (approx. 10% *w*/*w*) was incorporated into the composite material as a water repellent to reduce the starch’s water absorption, improving the material’s stability in humid environments without compromising its compostability [8]. Since each of the components in the compost has a different biodegradation potential, it was necessary to determine the biodegradability of the complete material, so the biodegradation test was carried out under composting conditions.

**Figure 2:**
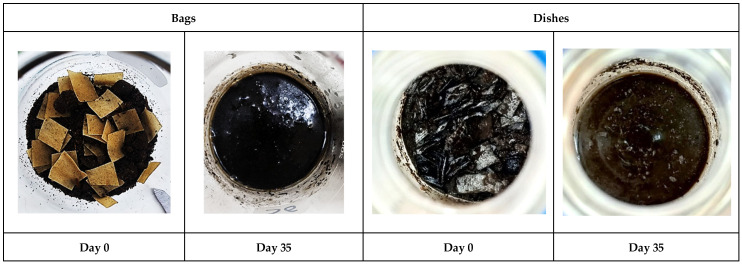
Evolution of the degradation of composite TPS-cassava bags and dishes under laboratory composting conditions.

The biodegradation of the composite TPS-cassava bags and dishes was evaluated in 35 days of composting conditions based on their evolved CO_2_ emission. Figure 2 shows the changes in each material and the degree of fragmentation between day 0 and day 35 of evaluation.

**Figure 3:**
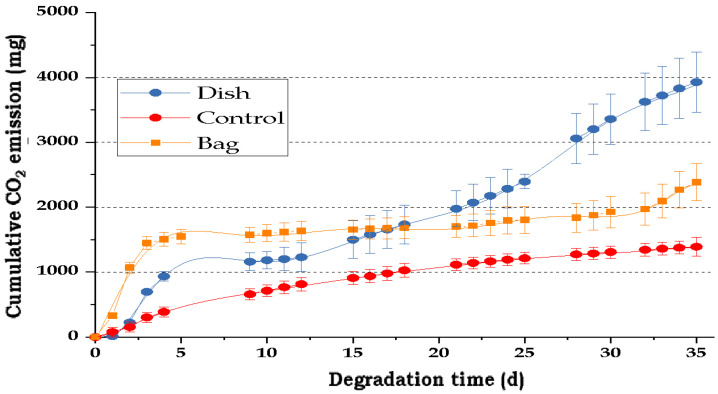
Cumulative CO_2_ evolution of composite TPS-cassava bags and dishes biodegradation over time. Control refers to blanks without biodegradable material.

**Figure 4:**
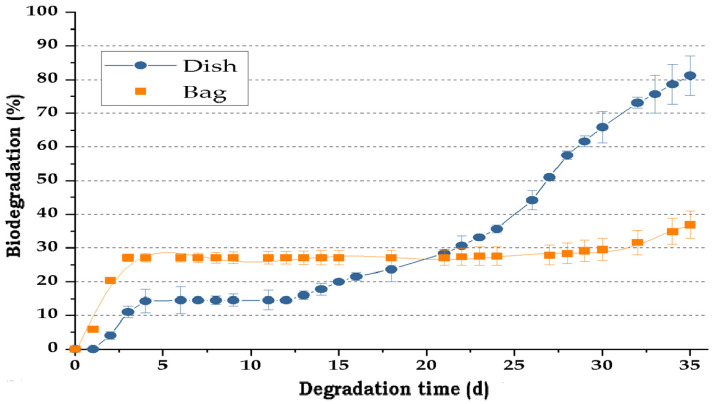
Aerobic biodegradation (% *w*/*w*) for the composite TPS-cassava bags and dishes over time.

Figure 3 illustrates the CO_2_ emissions of the bag and dish materials during their biodegradation process, using humus as a positive control. Figure 4 indicates the loss of starch, cellulose, and glycerol of those materials over time.

Controlled degradability assays indicated that after three months, biodegradable bags and dishes lost around 48% of their biomass, with no significant difference between products. Our field results were like the bag curve shown in Figure 3, indicating that there is a certain recalcitrancy of the biodegradable packages.

### 3.3. Accumulation of Non-Biodegradable Residues of TPS-Cassava/Plantain Leaves/Glycerol-Based and Plastic Packages

Cassava starch was, in both cases, the main carbon contributor to the composition of biodegradable packages (70% for bags and 32% for dishes). After starch, glycerol was the second most important contributor to C on biodegradable packages. Glycerol is crucial in converting starch into a biopolymer, enhancing the flexibility and durability of materials. Although citric acid is present in minimal quantities, its impact is significant due to the energy-intensive fermentation and purification required for its production, recommending the use of this from natural resources such as those extracted from fruits or residues. Comprehensive life cycle data for Span 80 is limited. Span 80 is an emulsifier essential for ensuring the uniform dispersion of components, enhancing the structural integrity and stability of the final product. Span 80 derives its emissions from sorbitol and oleic acid and their associated chemical production. Additional binders used, such as carboxymethylcellulose, which improves the dispersion of components such as waxes into glycerol, contribute 10% of total emissions, due to the CO_2_ produced during cellulose extraction (2.0-ton CO_2_/ton CMC), carboxylation with chloroacetic acid (0.8-ton CO_2_/ton CMC), and final purification (0.5-ton CO_2_/ton CMC). Pulverized banana fiber makes minimal C contributions due to the low energy input required for processing sun-dried banana leaves. This fiber reduces the overall footprint and recycles agricultural waste, sequestering carbon that would otherwise be released during natural decomposition. This evaluation of the source of materials highlights the importance of selecting those materials related to their C input as local sources, and new materials obtained from waste will reduce the amount of C in the final product.

A comparative analysis indicates that composite TPS-cassava bags and dishes have a significantly lower carbon footprint than traditional synthetic resins (Table 1). The carbon footprint of biodegradable bags was estimated at 1.47 kg CO_2_-eq per kilogram and 2.01 kg CO_2_-eq per kilogram for dishes, while non-degradable materials used traditionally for packaging productions are between 1.8 and 5.42 kg CO_2_-eq per kilogram (Table 2). The elevated CO_2_ emissions associated with synthetic resin production are due to their derivation from fossil fuels. Likewise, the treatment process for fossil-based plastics such as polystyrene and polypropylene often involves oxidation and incineration, the latter being highly polluting. A study has shown that burning plastics in a life cycle emits approximately 2.7 kg CO_2_-eq per kg of plastic burned, added to the 2.9 kg CO_2_-eq kg used in its production [44]. The extraction and processing of these raw materials are energy-intensive, leading to significant greenhouse gas emissions. Additionally, polymers considered biodegradable, such as PLA, can only be degraded by industrial composting under controlled conditions. These facilities exist only in certain developed countries and are not an alternative for developing countries such as Colombia. Therefore, PLA emissions were taken as total emissions without any reduction in the value reported associated with their degradability.

The manufacturing process for starch-derived products requires electrical energy for every kilogram of material produced (Table 3), with a total consumption of 23.98 kWh for biodegradable bag production and 29.23 kWh for biodegradable dish production. In bag production, the extrusion process, in which extruders, laminators, and pelletizers are used, represents the highest energy demand with 10 kWh (47% of the total demand), underlining the high energy required to transform natural materials into thermoplastics. There is a significant difference between extrusion and compression processes that are derived from different products and consume different energy. Thermocompression used to obtain biodegradable dishes only accounts for approximately 36% of the total energy consumption, which was, in this case, a medium consumption process compared to the extrusion process of the bags. The opposite occurs in intermediate processes: drying, pulverizing, and sieving starch in the production of bags represent 41.3% of the total energy required, while in the case of the dishes, the consumption of these intermediate processes requires 59.80% of the total energy consumption. These stages of processing significantly affect the final C footprint of the packages.

The amount of carbon emissions associated with the energy consumption of plastic package production is estimated at 26.45 kWh per bag and 28.24 kWh for dishes [9]. In the case of the biodegradable bags, in the way those are produced in Puerto Nariño locally, they are produced with a lower carbon footprint than traditional plastic bags, while in the case of dishes, biodegradable ones include a higher carbon footprint than plastic ones. This indicates that the process selected for packing production is critical in terms of the final carbon footprint of the product. However, not all the materials are suitable to be thermoplastified by the same technique, and the technique defined will depend on the final product expected and the feasibility of the biopolymer to be transformed by one or another technique.

Renewable energy sources, such as hydropower and wind energy, produce the lowest emissions due to their minimal reliance on fossil fuels and the sustainable nature of their energy generation (Table 4). Solar energy, which is a renewable option, shows slightly higher emissions primarily due to the life cycle impacts associated with the manufacturing and disposal of photovoltaic panels [45]. In contrast, fossil fuels always have high carbon footprint values: natural gas produces a moderate level of emissions, and petroleum has a high carbon footprint due to the energy-intensive processes of extraction, refining, and combustion. Coal, being the most carbon-intensive energy source, has the highest environmental impact, highlighting its inefficiency and substantial contribution to greenhouse gas emissions. The analysis made in this way underscores the crucial role of energy source selection in minimizing the carbon footprint of starch-based materials production.

The energy requirements for producing plastic and starch bags by extrusion processes are similar. For example, the energy consumption per kilogram for the processing of LDPE is 26.11 kWh [46], while the composite TPS-cassava bag production is 23.98 kWh. Interestingly, thermo-pressing of biodegradable dishes from this study (0.0025 kWh) is lower than the energy required to press and mold tree leaves into dishes (0.0086 kWh) and molding paper dishes (0.0027 kWh) [47]. This similarity is because the extrusion, granulation, and pressing equipment used to process thermoplastic polymers follow comparable mechanical and operating principles, resulting in overlapping energy consumption ranges. However, a key advantage of processing starch-based materials is the lower extrusion temperature required compared to plastic materials, which significantly reduces energy consumption. Fossil-based plastics require higher thermal conditions to melt and mold, while starch-based biopolymers can be processed at lower temperatures, reducing overall energy consumption and improving the sustainability of the manufacturing process. This lower thermal demand improves energy efficiency and contributes to lower associated carbon emissions, reinforcing the environmental advantages of starch-based alternatives over conventional plastic materials.

The carbon footprint required for transporting the composite TPS-cassava bag and dish production was evaluated by comparing two scenarios: transporting raw materials for local production to Puerto Nariño, Amazonas, and transporting finished plastic bags from Bogotá. The analysis considered only transport emissions from Bogotá to Puerto Nariño, as Bogotá is Colombia’s central distribution hub for industrial materials. Table 5 shows that for the local production of 10 tons of TPS-cassava/plantain leaves/glycerol-based bags, only 2.8 tons of raw materials require long-distance transport since cassava starch and plantain leaves were sourced locally. These materials were transported 1.095 km by air from Bogotá to Leticia and 80 km by river from Leticia to Puerto Nariño, resulting in total emissions of 1852.6 kg CO_2_, with air transport contributing 99.6%. These emphasized the need to realize the high impact caused by using imported biodegradable packages when a country does not develop its capacity to produce them locally. The simple use of biodegradable packages is not enough to be less environmentally harmful. In the case of Colombia, most biodegradable packages that are offered to customers come from Asia, having a high C footprint when they arrive in the customer’s hands. If 10 tons of finished plastic bags or dishes were transported along the same route used for plastic packages, emissions would rise to 6616.7 kg CO_2_, with air freight again being the dominant factor. Thus, the composite TPS-cassava bag production benefits from local sourcing, significantly reducing transportation-related emissions. Local production reduces transport emissions by 72% to 82% compared to importing finished plastic bags.

Regarding the degradability of the composite TPS-cassava packages, it was observed a more noticeable increase during the final days (25–35), the materials showed significant CO_2_ release during the first five days (30–15%), with 50–30% of the total cumulative emissions of bags and dishes, respectively, and ended with 40–80% of total decomposition. As indicated before, after a couple of weeks, there is a kind of recalcitrance of the bio-packages to be degraded due to the presence of natural materials resistant to degradation such as wax (215 mg CO_2_/d for dishes and 310 mg CO_2_/d for bags), which also shows high hydrophobicity, reducing the quantity of water that can be incorporated to the biopolymer, affecting the microbial activity [48]. In addition to wax, there are other materials in the recipe of biodegradable bags and dishes, such as LDPE and Span 80, used to reduce the susceptibility of the packages to moisture, with low biodegradability, and that need more time to be completely degraded. These materials make the packages durable and limit the amount of C released to the environment in short periods. It was also demonstrated that the patterns of degradation found in lab conditions were similar under natural conditions, indicating that predictions might be close to the real way in which the bio-package degradation occurs, under these extreme climate conditions with high environmental temperature and moisture.

The accumulation of residues in composite TPS-cassava bags with only 2% of non-biodegradable content corresponds to an annual waste generation rate of 298.5 kg per year based on a hypothetical production of 1 million bags. In comparison, for the same LDPE bag generation, the annual accumulation of non-biodegradable waste will be 9950 kg, given the slower biodegradation rate of this polymer, as shown in Table 6 [32]. Furthermore, it is also necessary to consider that the degradation of conventional plastic materials releases toxic compounds inherent to their composition, which might include phthalates, bisphenol A (BPA), and heavy metals commonly used to enhance plastic’s flexibility, durability, or color. These chemicals leach into the environment during the slow degradation of plastic, posing significant risks to ecosystems and human health [49]. The combination of persistent non-biodegradable materials and toxic chemical leaching exacerbates the environmental burden of conventional plastics, emphasizing the urgency of adopting safer, biodegradable alternatives.

As it is presented in Table 6, the amount of non-biodegradable residues is higher in conventional plastic packages. Even when it is assumed that 2% of non-biodegradable materials are added to biopackage production, the transition from plastics to bioplastics will offer a reduction in the volume and weight of residues, and a reduction in micro- and nano-plastics released to the environment. This qualitative change is relevant as, until today, there are few effective methods for managing microplastic residuals [50], and it will be significant for areas where the waste management is deficient, as occurs in developing countries.

## 4. Conclusions

The carbon footprint is an accurate tool to evaluate the real impact that a product has on the environment. In this work, bags and dishes made from thermoplastic cassava, plantain leaves as natural reinforcing fibers, and glycerol as a plasticizer were considered as a case study. These components were selected due to their local availability and biodegradability. The mechanical and environmental performance of the packages developed in this study is therefore directly linked to the properties of these materials. This carbon footprint might include the life cycle of the product and the permanence of its residues.

The nature of a product (and whether this is biodegradable or not) is not enough to deduce its carbon footprint. In the case of composite TPS-cassava bags and dishes, it was revealed that the importance of the raw-material extraction, conditioning, and processing in the same area is in order to reduce the carbon footprint associated with the transportation between stages of the manufacturing process. This case study also demonstrates that it is possible to develop local starch-based biodegradable packages resistant to extreme conditions, such as high environmental temperature and moisture, selecting natural hydrophilic ingredients, and defining low-carbon energy sources, such as renewable sources of energy. The biodegradable nature of the materials used to make this type of bio-packaging is insufficient to determine the carbon footprint of its manufacturing process, which can make it difficult to select methodologies that lead to a lower overall environmental impact. After marketing, providing adequate information to consumers can lead to a lower carbon footprint if the final disposal is appropriate, promoting the best conditions for biodegradation.

## Figures and Tables

**Table 1:** Carbon footprint contribution of raw materials for composite TPS-cassava packages.

Material	Mass Fraction	Factor of Emission (kg CO_2_-eq/kg)	Source	Partial Emission (kg CO_2_-eq/kg)	Emission (kg CO_2_-eq/FU)
**Composite TPS-cassava bags**					
Cassava starch	0.71	1.03	[26]	0.726	3.63
Glycerol	0.23	2.00	[34]	0.460	2.3
Beeswax	0.02	0.41	[35]	0.008	0.04
Citric acid (EEUU)	0.01	19.32	[26]	0.193	0.965
Span 80	0.01	4.00	[26]	0.020	0.1
LDPE	0.02	3.43	[36]	0.069	0.345
Powered plantain leaves	0.01	0.002	[37]	0.00002	0.0001
**Total carbon footprint/kg bag material**				**1.476**	**7.38**
**Composite TPS-cassava dishes**					
Cassava starch	0.322	1.03	[26]	0.94	195.52
Glycerol	0.086	2.00	[34]	0.488	101.50
Beeswax	0.032	0.41	[35]	0.037	3.76
Citric acid (EEUU)	0.010	2.1	[26]	0.063	13.10
Span 80	0.032	4.00	[26]	0.364	75.71
carboxymethylcellulose (CMC)	0.021	3.40	[26]	0.207	43.06
Powered plantain leaves	0.129	0.002	[37]	0.00074	0.15
**Total carbon footprint/kg dish material**				**2.099**	**436.60**

**Table 2:** Carbon emission factors of common synthetic and biodegradable resins.

Resin	Factor of Emission (kg CO_2_-eq/kg)	Emission (kg CO_2_-eq/FU)
Polyethene (PE)	**3.43**	**17.15**
Polypropylene (PP)	**4.72**	**23.60**
Polyvinyl chloride (PVC)	**5.42**	**27.10**
Polylactic acid (PLA)	**1.8–3.3**	**12.75**
Polyhydroxyalkanoates (PHAs)	**2.5–4.5**	**17.50**

Adapted from [36,38].

**Table 3:** Energy consumption of equipment required for composite TPS-cassava bag and dish production. Values of energy consumption for plastic materials were extracted from Chen et al. [11], who used the same FU for their estimations.

Stage	Activity to Perform	Equipment	Energy Consumption (kW/h)
Composite TPS-cassava bags			
Raw Material Reception	Weight validation of raw materials	Industrial floor scale	0.500
Material Conditioning	Starch drying	12-tray dehydrator	2.240
	Starch pulverization	Pulverizer mill	3.000
	Starch sieving ≤400 µm	Vibratory sieve	3.500
	Starch moisture validation	Thermogravimetric balance	1.000
Mixing	Material mixing	Mixer	2.240
Pelletizing	Pellet production	Pelletizing extruder	5.000
Sheet Extrusion	Extrusion	Sheet extruder	5.000
Bag Precut	Precutting rolls	Bag precutter	1.500
**Estimated total energy consumption for biocomposite bag production**			**23.980**
**Composite TPS-cassava dishes**			
Raw Material Reception	Weight validation of raw materials	Industrial floor scale	0.500
Material Conditioning	Starch/plantain leaves drying	12-tray dehydrator	4.480
	Starch/plantain leaves pulverization	Pulverizer mill	6.000
	Starch/plantain leaves sieving ≤100 µm	Vibratory sieve	7.000
Mixing	Material melting/mixing	Mixer	6.250
Pressing	Material pressing	Hydraulic press	5.000
**Estimated total energy consumption for biocomposite dish production**			**29.230**

**Table 4:** Carbon emissions per energy source for one hour of composite TPS-cassava bag and dish production.

Energy Source	CO_2_ Emission Factor (kg CO_2_/kWh) *	Total CO_2_ Emissions per Hour (Bags) (kg CO_2_/h)	Emissions per FU (Bags) (kg CO_2_/FU)	Total CO_2_ Emissions per Hour (Dishes) (kg CO_2_/h)	Emissions per FU (Dishes) (kg CO_2_/FU)
Hydropower	0.004	0.096	0.024	0.117	1.218
Wind	0.011	0.264	0.066	0.321	3.344
Solar	0.041	0.983	0.246	1.200	12.50
Natural Gas	0.93	22.301	5.575	27.184	283.16
Petroleum	1.17	28.057	7.014	34.200	356.25
Coal	1.689	40.502	10.126	49.400	514.58

* Source: [39].

**Table 5:** Transport emissions by mode and material type for composite TPS-cassava bags and dishes.

Transport Mode	Fuel Type	Emission Factor (kg CO_2_/ton-km) *	Distance (km)	Mass Transported—Raw Materials (Tons)	Emissions—Raw Materials Transported (kg CO_2_)	Emissions per FU (kg CO_2_/FU)	Mass Transported—LDPE Bags (Tons)	Emissions-LDPE Bags (kg CO_2_)	Emissions per FU—LDPE Bags (kg CO_2_/FU
**Air Transport (Bogotá → Leticia)**	**Jet Fuel**	0.602	1095	2.8	1845.732 (bags)	0.923 (bags)	10	6591.9	3.30
				1.81	1193.134 (dishes)	24.82 (dishes)			
**River Transport (Leticia → Puerto Nariño)**	**Gasoline**	0.031	80	2.8	6.944 (bags)	0.0035 (bags)	10	24.8	0.01
				1.81	4.488 (dishes)	0.093 (dishes)			
**Composite TPS-cassava packages**				**Total Emissions (kg CO_2_)**	1852.676 (bags)	0.926 (bags)	Total emissions (kg CO_2_)	6616.7	3.31
					1197.623 (dishes)	24.91 (8dishes)			

* Source: [19,40].

**Table 6:** Theoretical comparison of non-biodegradable component degradation between the evaluated composite TPS-cassava packages and conventional LDPE bags.

Material Type	Annual Bag Consumption (Units)	Weight per Bag (kg)	Non-Biodegradable Content (%)	Non-Biodegradable Residues (kg)	Degradation Rate of Non-Biodegradable Residues (kg/Year) [32]	Non-Biodegradable Residues Remaining After a Year (kg)
Composite TPS-cassava Bags	1,000,000	0.015	2%	300	0.005	298.5
Conventional LDPE Bags	1,000,000	0.010	100%	10,000	0.005	9950

## Data Availability

The original contributions presented in this study are included in the article. Further inquiries can be directed to the corresponding author.

## References

[1] Geyer R., Jambeck J.R., Law K.L. (2017). Production, Use, and Fate of All Plastics Ever Made. Sci. Adv..

[2] Stegmann P., Daioglou V., Londo M., Van Vuuren D.P., Junginger M. (2022). Plastic Futures and Their CO_2_ Emissions. Nature.

[3] Jayavel S., Govindaraju B., Michael J.R., Viswanathan B. (2024). Impacts of Micro and Nanoplastics on Human Health. Bull. Natl. Res. Cent..

[4] Sharma S., Chatterjee S. (2017). Microplastic pollution, a threat to marine ecosystem and human health: A short review. Environ. Sci. Pollut. Res..

[5] Bressanin J.M., Sampaio I.L.D.M., Geraldo V.C., Klein B.C., Chagas M.F., Bonomi A., Filho R.M., Cavalett O. (2022). Techno-Economic and Environmental Assessment of Polylactic Acid Production Integrated with the Sugarcane Value Chain. Sustain. Prod. Consum..

[6] Lee C.H., Sapuan S.M., Ilyas R.A., Lee S.H., Khalina A. (2020). Development and Processing of PLA, PHA, and Other Biopolymers. Advanced Processing, Properties, and Applications of Starch and Other Bio-Based Polymers.

[7] Agarwal S., Singhal S., Godiya C.B., Kumar S. (2023). Prospects and Applications of Starch-Based Biopolymers. Int. J. Environ. Anal. Chem..

[8] Reis M.O., Olivato J.B., Bilck A.P., Zanela J., Grossmann M.V.E., Yamashita F. (2018). Biodegradable Trays of Thermoplastic Starch/Poly (Lactic Acid) Coated with Beeswax. Ind. Crops Prod..

[9] Ferreira D.C.M., Molina G., Pelissari F.M. (2020). Biodegradable Trays Based on Cassava Starch Blended with Agroindustrial Residues. Compos. Part B Eng..

[10] Chen G., Li J., Sun Y., Wang Z., Leeke G.A., Moretti C., Cheng Z., Wang Y., Li N., Mu L. (2024). Replacing Traditional Plastics with Biodegradable Plastics: Impact on Carbon Emissions. Engineering.

[11] Chen Y., Awasthi A.K., Wei F., Tan Q., Li J. (2021). Single-Use Plastics: Production, Usage, Disposal, and Adverse Impacts. Sci. Total Environ..

[12] Zheng J., Suh S. (2019). Strategies to Reduce the Global Carbon Footprint of Plastics. Nat. Clim. Change.

[13] Meng F., Brandão M., Cullen J.M. (2024). Replacing Plastics with Alternatives Is Worse for Greenhouse Gas Emissions in Most Cases. Environ. Sci. Technol..

[14] Essel R.E., Engel L., Carus M. (2015). Sources of Microplastics Relevant to Marine Protection in Germany.

[15] Xu Y., Chan F.K.S., Johnson M.F., He J., Stanton T., Joo S.H. (2022). A Review of Microplastic Pollution Characteristics in Global Urban Freshwater Catchments. Advances in Human Services and Public Health.

[16] Boucher J., Friot D. (2017). Primary Microplastics in the Oceans: A Global Evaluation of Sources.

[17] Jambeck J.R., Geyer R., Wilcox C., Siegler T.R., Perryman M., Andrady A., Narayan R., Law K.L. (2015). Plastic Waste Inputs from Land into the Ocean. Science.

[18] Lebreton L.C.M., Van Der Zwet J., Damsteeg J.-W., Slat B., Andrady A., Reisser J. (2017). River Plastic Emissions to the World’s Oceans. Nat. Commun..

[19] Boucher J., Dubois C., Kounina A., Puydarrieux P. (2019). Review of Plastic Footprint Methodologies: Laying the Foundation for the Development of a Standardised Plastic Footprint Measurement Tool.

[20] Keyes A., Saffron C.M., Manjure S., Narayan R. (2024). Biobased Compostable Plastics End-of-Life: Environmental Assessment Including Carbon Footprint and Microplastic Impacts. Polymers.

[21] Olanrewaju O.I., Enegbuma W.I., Donn M. (2024). Challenges in Life Cycle Assessment Implementation for Construction Environmental Product Declaration Development: A Mixed Approach and Global Perspective. Sustain. Prod. Consum..

[22] Walker S., Rothman R. (2020). Life Cycle Assessment of Bio-Based and Fossil-Based Plastic: A Review. J. Clean. Prod..

[23] Lee K.-M., Inaba A. (2004). Life Cycle Assessment: Best Practices of International Organization for Standardization (ISO) 14040 Series.

[24] Garavito J., Castellanos-González S., Peña-Venegas C.P., Castellanos D. (2025). Development and Characterization of Reinforced Flexible Packaging Based on Amazonian Cassava Starch Through Flat Sheet Extrusion. Preprint.

[25] Henao Cardona L.F., Castillo Aguilar M.C., Moreno Méndez J.O., Marín López C., Maldonado A., Castrodelrío Ceballos J.A. (2015). Guía Para La Formulación, Implementación, Evaluación, Seguimiento, Control y Actualización de Los Planes de Gestión Integral de Residuos Sólidos (PGIRS).

[26] CarbonCloud CarbonData Climate Footprint Data on Food Products. https://www.carboncloud.io/carbondata/.

[27] Chen F., Lei J., Liu Z., Xiong X. (2025). A Comparative Study on the Average CO_2_ Emission Factors of Electricity of China. Energies.

[28] ECTA, CEFIC, ECIC (2011). Guidelines for Measuring and Managing CO2 Emission from Freight Transport Operations.

[29] (2021). Standard Test Method for Determining Aerobic Biodegradation of Plastic Materials Under Controlled Composting Conditions, Incorporating Thermophilic Temperatures.

[30] Withana P.A., Yuan X., Im D., Choi Y., Bank M.S., Lin C.S.K., Hwang S.Y., Ok Y.S. (2025). Biodegradable Plastics in Soils: Sources, Degradation, and Effects. Environ. Sci. Process. Impacts.

[31] Yu Y., Flury M. (2024). Unlocking the Potentials of Biodegradable Plastics with Proper Management and Evaluation at Environmentally Relevant Concentrations. npj Mater. Sustain..

[32] Zhang Z., Fan X., Zhang R., Pan X., Zhang X., Ding Y., Liu Y. (2025). Biodegradation characterization and mechanism of low-density polyethylene by the enriched mixed-culture from plastic-contaminated soil. J. Hazard. Mater..

[33] Channab B.-E., El Idrissi A., Zahouily M., Essamlali Y., White J.C. (2023). Starch-Based Controlled Release Fertilizers: A Review. Int. J. Biol. Macromol..

[34] Quispe C.A.G., Coronado C.J.R., Carvalho J.A. (2013). Glycerol: Production, Consumption, Prices, Characterization and New Trends in Combustion. Renew. Sustain. Energy Rev..

[35] Maglaya I. (2020). Life Cycle Analysis of Nonpetroleum Based Wax. Master’s Thesis.

[36] An J., Wu F., Wang D., You J. (2022). Estimated Material Metabolism and Life Cycle Greenhouse Gas Emission of Major Plastics in China: A Commercial Sector-Scale Perspective. Resour. Conserv. Recycl..

[37] Veliz K., Chico-Santamarta L., Ramirez A.D. (2022). The Environmental Profile of Ecuadorian Export Banana: A Life Cycle Assessment. Foods.

[38] Asunis F., De Gioannis G., Francini G., Lombardi L., Muntoni A., Polettini A., Pomi R., Rossi A., Spiga D. (2021). Environmental Life Cycle Assessment of Polyhydroxyalkanoates Production from Cheese Whey. Waste Manag..

[39] Pedersen S.K. Comparing CO_2_ Emissions from Different Energy Sources. https://www.cowi.com/news-and-press/news/2023/comparing-co2-emissions-from-different-energy-sources/.

[40] Core Writing Team IPCC (2015). Climate Change 2014: Synthesis Report. Contribution of Working Groups I, II and III to the Fifth Assessment Report of the Intergovernmental Panel on Climate Change.

[41] Jumaidin R., Diah N.A., Ilyas R.A., Alamjuri R.H., Yusof F.A.M. (2021). Processing and Characterisation of Banana Leaf Fibre Reinforced Thermoplastic Cassava Starch Composites. Polymers.

[42] Ma X., Yu J., Kennedy J.F. (2005). Studies on the Properties of Natural Fibers-Reinforced Thermoplastic Starch Composites. Carbohydr. Polym..

[43] Wang C., Li F., Wang L., Li J., Guo A., Zhang C., Liu P. (2015). Research on Thermoplastic Starch and Different Fiber Reinforced Biomass Composites. RSC Adv..

[44] Filho W.L., Barbir J.E., Carpio-Vallejo A.D., Voronova V. (2025). Decarbonising the plastic industry: A review of carbon emissions in the lifecycle of plastics production. Sci. Total Environ..

[45] Mehedi T.H., Gemechu E., Kumar A. (2022). Life cycle greenhouse gas emissions and energy footprints of utility-scale solar energy systems. Appl. Energy.

[46] Marczak H. (2022). Energy Inputs on the Production of Plastic Products. J. Ecol. Eng..

[47] Korbelyiova L., Malefors C., Lalander C., Wikström F., Eriksson M. (2021). Paper vs Leaf: Carbon Footprint of Single-Use Plates Made from Renewable Materials. Sustain. Prod. Consum..

[48] Zhu S., Li M., Qian T., Chen J., Pan T. (2025). Influence of Surfactants on Interfacial Microbial Degradation of Hydrophobic Organic Compounds. Catalysts.

[49] Yoezer N., Gurung D.B., Wangchuk K. (2023). Environmental Toxicity, Human Hazards and Bacterial Degradation of Polyethylene. Nat. Environ. Pollut. Technol..

[50] Thacharodi A., Hassan S., Meenatchi R., Bhat M.A., Hussain N., Arockiaraj J., Ngo H.H., Sharma A., Nguyen H.T., Pugazhendhi A. (2024). Mitigating microplastic pollution: A critical review on the effects, remediation, and utilization strategies of microplastics. J. Environ. Manag..

